# Association Between *VDR* FokI Polymorphism and Intervertebral Disk Degeneration

**DOI:** 10.1016/j.gpb.2015.11.003

**Published:** 2016-01-06

**Authors:** Jian Zhao, Mingyuan Yang, Jie Shao, Yushu Bai, Ming Li

**Affiliations:** Department of Orthopaedics, Changhai Hospital, Second Military Medical University, Shanghai 200438, China

**Keywords:** FokI polymorphism, rs2228570, Vitamin D receptor, Disk degeneration, Intervertebral disk disease

## Abstract

Intervertebral disk degeneration (IDD) is strongly associated with genetic predisposition and environmental susceptibility. Several studies been conducted to investigate the potential association between IDD and **FokI polymorphism** located in the gene encoding the **vitamin D receptor** (VDR), and inconsistent conclusions had been reached among different ethnic populations. In order to assess the association between the **FokI polymorphism** and the risk of IDD, we performed a comprehensive and systematic meta-analysis. Candidate articles were retrieved from PubMed, EMBASE, China National Knowledge Infrastructure (CNKI), and China Biology Medical (CBM) with strict inclusion criteria in January 2015. Among the 54 articles that were retrieved, only eight studies met the inclusion criteria. The pooled data analysis based on allele contrast, homozygote, heterozygote, dominant, and recessive models revealed no significant correlation between the **FokI polymorphism** and the risk of IDD. However, when stratified by ethnicity, significant associations were detected for Hispanics based on allele contrast (OR = 1.395, 95% CI = 1.059–1.836, *P* = 0.018), homozygote (OR = 1.849, 95% CI = 1.001–3.416, *P* = 0.049), heterozygote (OR = 1.254, 95% CI = 1.049–1.498, *P* = 0.013), and dominant (OR = 1.742, 95% CI = 1.174–2.583, *P* = 0.006) models, and for Asians using the dominant model (OR = 1.293, 95% CI = 1.025–1.632, *P* = 0.030), whereas there is no significant association detected for Caucasians. In conclusion, **FokI polymorphism** is not generally associated with IDD, but there is increased risk for IDD in Hispanics and Asians carrying FokI allele T.

## Introduction

The intervertebral disk functions as a fibrocartilaginous cushion pad against mechanical load from the coronal, sagittal, and transverse planes. Pathophysiological intervertebral disk degeneration (IDD) is primarily characterized by deterioration and dysfunction of the annulus fibrous and nucleus pulpous, resulting in a range of symptoms such as chronic neck pain, lower back pain, and upper and lower extremity pain [1], [2]. Several etiological factors of IDD, such as advanced age, gender, obesity, hard physical labor, and tobacco consumption, have recently been identified [3]. Mechanistic studies of IDD pathogenesis also pointed out that IDD is strongly linked with genetic heterogeneity and environment susceptibility. Likewise, family pedigree analysis [4] and candidate gene studies [5] have found that genetic variants can affect the susceptibility to IDD.

Recently, several institutions have reported diverse genetic biomarkers related to IDD, including the polymorphisms in the gene encoding vitamin D receptor (VDR) [5]. VDR is a nuclear receptor, which is translocated from cytoplasm to nucleus upon activation by binding of its ligand 1-α-25-dihydroxyvitamin D3 (1-α-25(OH) 2D3) [6]. Subsequent interactions with transcriptional corepressor or coactivator factors are critical for nuclear vitamin D signaling pathway [6]. The human *VDR* gene, located on chromosome 12 (12q12–q14), spans over 105 kb with a remarkably large promoter [7]. Over 100 potential restriction fragment length polymorphism (RFLP) sites have been found [8]. Initially identified to be a gene predisposing to IDD in 1998 [9], *VDR* is now considered to be one of the genetic biomarkers associated with IDD [6]. In this context, there have been an increasing number of studies reporting a correlation between IDD and *VDR* gene polymorphisms, such as *Taq*I (rs731236), FokI (rs2228570), and *Apa*I (rs7975232) [10].

The FokI polymorphism (C to T transition) is located in the start codon of the *VDR* gene, which leads to an alternative translation start site [7]. The wild type allele C (F allele) of this polymorphism site produces a receptor composed of 424 amino acid residues, whereas the variant allele T (f allele) encodes a product of 427 amino acid residues [8]. Several studies have investigated the functions of VDR proteins of different lengths, and reported that the shorter polypeptide is of higher efficiency to couple with the transcription factor II B (TFIIB) and leads to a higher transcriptional rate of vitamin dependent genes [11], [12].

In addition, many studies [13], [14], [15], [16], [17], [18], [19], [20], [21] have investigated the association between FokI polymorphism and IDD. However, inconsistent results [13], [14], [15], [16], [17], [18], [19], [20], [21] were obtained among different ethnic populations. For instance, a significant association of *VDR* FokI T/C transition with IDD was reported in Hispanics [13] and Caucasians [21]. However, Chen et al. [20] reported that there was no significant association between *VDR* FokI T/C transition and IDD in Chinese. Furthermore, Xu et al. performed a meta-analysis [22] in Caucasians and Asians with 425 cases and 608 controls and concluded the absence of any significant association between the *VDR* FokI polymorphism and IDD. In order to assess the association between the FokI polymorphism and the risk of IDD, our meta-analysis systematically and comprehensively evaluated the evidence accumulated to date with relatively larger sample size and higher statistical power.

## Results

### Search results and study characteristics

**Figure 1:**
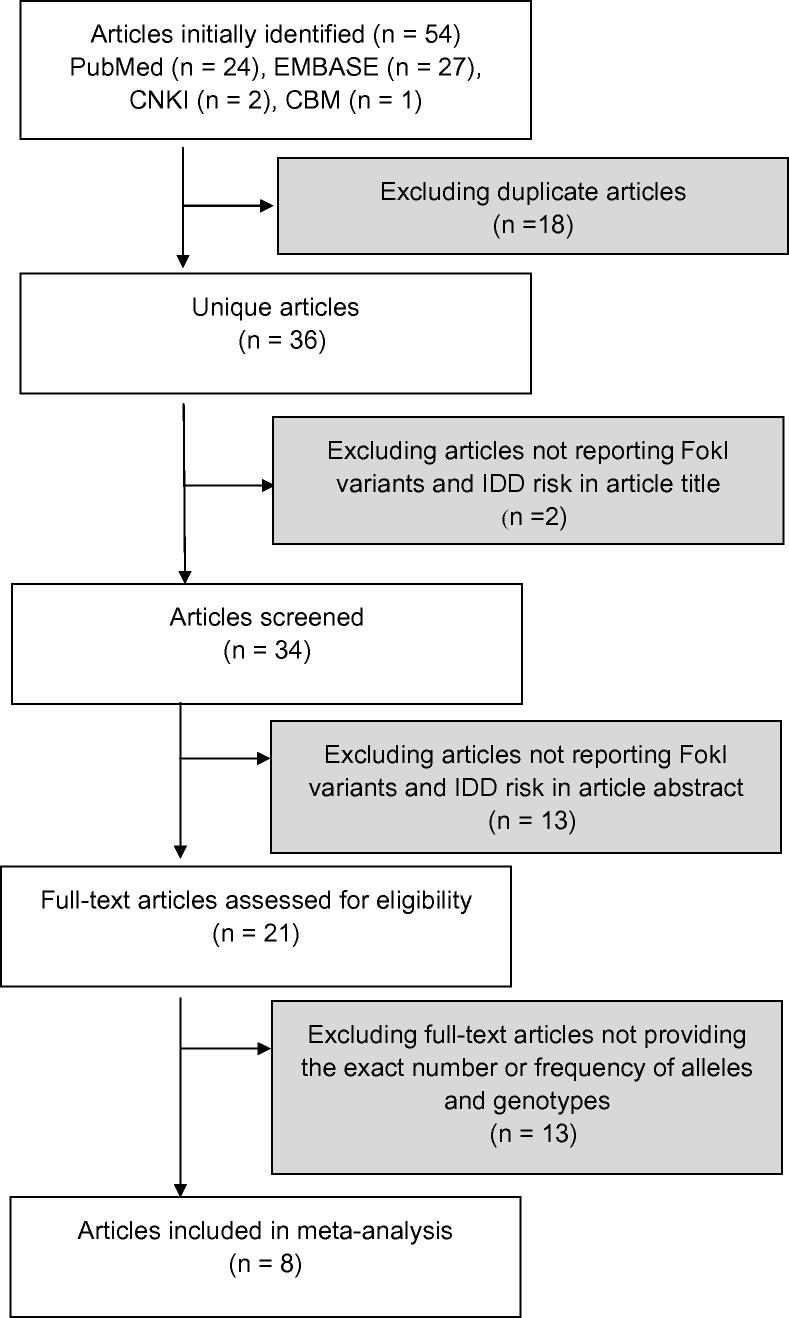
**Flow chart illustrating the selection of studies for meta-analysis**

We retrieved 54 articles through literature search of PubMed, EMBASE, China National Knowledge Infrastructure (CNKI), and China Biology Medical (CBM). After excluding articles such as duplicates and non-case controlled studies (see Materials and methods for detailed inclusion and exclusion criteria), eight studies were finally included in the meta-analysis (Figure 1). Among those studies, only one study was conducted on Asians [20], two on Hispanics [14], [15], and the remaining 5 studies were on Caucasians [13], [16], [17], [18], [19]. In total, there were 1118 cases and 1073 controls included in this meta-analysis. All DNA samples were extracted from leukocytes [13], [14], [15], [16], [17], [18], [19], [20], [21], and IDD was diagnosed by magnetic resonance imaging (MRI) with or without a computed tomography (CT) scan. The descriptive characteristics of the eligible studies are detailed in Table 1. The genotype distributions in the control groups agreed with Hardy–Weinberg equilibrium (HWE).

### Quantitative data analysis

Overall, this meta-analysis did not detect any significant correlation between the *VDR* FokI polymorphism and the increased susceptibility to IDD in the pooled population. The results were obtained based on different evaluation models, including allele contrast (T *vs*. C, OR = 1.023, 95% CI = 0.898–1.166, *P* = 0.728), homozygote (TT *vs*. CC, OR = 1.087, 95% CI = 0.806–1.443, *P* = 0.611), heterozygote (TC *vs*. CC, OR = 1.204, 95% CI = 0.982–1.476, *P* = 0.075), dominant (TC/TT *vs*. CC, OR = 1.166, 95% CI = 0.961–1.414, *P* = 0.119), and recessive (TT *vs*. TC/CC, OR = 0.853, 95% CI = 0.676–1.078, *P* = 0.183) models.

When stratified by ethnicity, however, significant associations were detected in the allele contrast (OR = 1.395, 95% CI = 1.059–1.836, *P* = 0.018), homozygote (OR = 1.849, 95% CI = 1.001–3.416, *P* = 0.049), heterozygote (OR = 1.254, 95% CI = 1.049–1.498, *P* = 0.013), and dominant (OR = 1.742, 95% CI = 1.174–2.583, *P* = 0.006) models for Hispanics, and in the dominant model (OR = 1.293, 95% CI = 1.025–1.632, *P* = 0.030) for Asians. On the other hand, no statistical significance was revealed in Caucasians. The pooled ORs and 95% CI based on the allele contrast, heterozygote, homozygote models dominant and recessive models are listed in Table 2.

### Sensitive analysis and publication bias

We next evaluated the sensitivity in this meta-analysis. No matter which article was rejected, the pooled results did not vary remarkably for the allele contrast model (Table 3) and the other four models. Therefore, the sensitivity of this study was guaranteed, indicating that data in this meta-analysis has relative stability and credibility. Egger’s test also confirmed that there was no significant publication bias in the allelic contrast (*P* = 0.151), homozygote (*P* = 0.540), heterozygote (*P* = 0.223), dominant (*P* = 0.426), and recessive models (*P* = 0.154).

## Discussion

The vitamin D endocrine system is involved in a range of biochemical processes [23]. Disorder of vitamin D metabolism can lead to bone metabolism dysfunction, calcium loss, and cartilage degeneration [24], while a persistent defect in vitamin D endocrine system was strongly involved in the aging of skeletal muscles such as paravertebral muscles [5]. VDR, as the specific anchor of vitamin D, plays an important role in the vitamin D endocrine system [25]. Sanders K et al. [26] found that transcription of the *VDR* gene was downregulated in skeletal muscle of the aged people. More important, *VDR* gene variation is highly associated with dysfunctions of vitamin D metabolism [3]. The T/C transition polymorphism in the *VDR* gene can cause alteration in the structure of the VDR protein [5]. Moreover, variation is strongly associated with different abilities of the VDR protein to bind TFIIB, leading to divergent gene transcription coupled with VDR [27], [28]. The shorter VDR protein consisting of 424 amino acid residues has a higher efficacy to activate TFIIB [29]. Therefore, polymorphism that affects the VDR protein length can exert heavy influence on the functions of the vitamin D metabolism system. Taken together with the aforementioned characteristics, this polymorphic site may have significant correlation with the core pathophysiological mechanism of IDD [8], [20].

A series of studies [13], [14], [15], [16], [17], [18], [19], [20], [21] and a meta-analysis [22] were performed to investigate the association between the polymorphism and predisposition to IDD among different ethnic populations. However, the previous meta-analysis only included five studies [16], [17], [18], [19], [21], limited by the studies available. Our current meta-analysis included four new studies [13], [14], [15], [20] by collecting articles published in Chinese and English, whereas one study [21] included in the previous meta-analysis was excluded because control group failed to meet the HWE requirement. In addition, the MRI images of control intervertebral disks had to strictly meet the criteria of normal shape, no horizontal bands, and easy distinction of the nucleus and annulus. Therefore, the larger study population and the strict inclusion criteria employed in the current study guaranteed a relatively strong statistical power. Overall, the pooled results suggest that there was no significant association between the polymorphism and IDD. Nonetheless, subgroup analysis found significant associations at the ethnicity level, where Hispanics and Asians showed significant association using selected models, but Caucasians did not. Each ethnic group may have differential genetic background and is bound to be exposed to different environmental factors as well, which leads to different susceptibilities to diseases, such as IDD. Factors like socioeconomic status, occupational load, cultural background, lifestyle, and body mass index (BMI) can account for inconsistent results among different ethnic groups [2], [14], [21]. Additionally, identified and unidentified genes can affect the statistical power, especially when gene–gene interactions, combined with gene–environment interactions are taken into account.

Although the current meta-analysis shows prominent strength of more cases and controls, the limitations of this study must also be taken into serious consideration. First, the calculations of pooled ORs combined with 95% CI were based on the original articles, without adjusting for BMI, age, gender, hard physical labor, tobacco consumption, *etc*. Additionally, it was difficult to adopt an adjustment model to eliminate these interfering factors efficiently, because there are too many covariates, compared with relatively small sample size. Second, the diagnoses of IDD were not identical, especially when it comes to the different clinical manifestations. Third, only one study [20] was conducted on Asians and two [13], [15] on Hispanics, which (coupled with the small sample size) might misrepresent the underlying associations. Finally, due to the language limitation, we only included the literature published in Chinese and English, and inevitably missed relevant studies written in other languages. Therefore, large-scale and well-designed research analyses are still very much needed to identify the potential associations among different ethnic populations.

## Conclusion

In conclusion, after comprehensively and systematically evaluating the available literature published in Chinese or English, our meta-analysis suggests that allele T at FokI polymorphism site is highly associated with IDD in Hispanics and Asians, but not in Caucasians. Given the aforementioned limitations of this study, further well-designed studies are needed to validate the correlation between the FokI polymorphism and IDD risk and explore the underlying mechanisms.

## Materials and methods

### Literature search strategy

For comprehensive evidence retrieval, we searched the candidate articles in several electronic bibliographical databases, including PubMed, EMBASE, CNKI, and CBM literature databases on January 15, 2015 without any limitation. The keywords for searching are “vitamin D receptor OR VDR” OR FokI, “polymorphisms OR single nucleotide polymorphism OR SNP OR variation”, and “intervertebral disk degeneration OR intervertebral degenerative disease OR IDD”. In order to obtain more studies, we retrieved the reference lists of the selected studies, reviews, and meta-analyses. The literature search was performed by two authors, and any conflict was resolved through discussion.

### Inclusion and exclusion criteria

Articles included in this meta-analysis had to meet the following criteria: (1) investigating the correlation between the polymorphism and the risk of IDD, (2) case–control design, (3) providing the exact number or frequency of alleles and genotypes, (4) distribution of genotype frequencies in control groups following HWE, and (5) publication in English or Chinese, and (6) having the larger (or largest) sample size if more than one papers published are based on the same population. Exclusion criteria include (1) reviews or meta-analysis or non-case-controlled design, (2) the exact number or frequency of alleles and genotypes not available, (3) genotype frequencies in controls not following HWE, and (4) duplicated reports based on the same population.

### Data extraction

After eligible articles were identified, data were extracted by two independent authors. The following information was extracted from the articles: name of the first author, year of publication, country of origin, ethnicity on which the analysis was conducted, numbers of cases and controls, and frequency of genotypes in case and control groups. Ethnicities were divided into Asians, Caucasians, and Hispanics.

### Data meta-analysis

Chi-square analysis was employed to investigate the potential deviation of HWE and *P* > 0.05 meant that the control group satisfied HWE. Pooled ORs combined with 95% CI were calculated to detect the potential correlation between this polymorphism and the IDD based on allelic effect (T *vs*. C), homozygote (TT *vs*. CC), heterozygote (TC *vs*. CC), dominant (TC/TT *vs*. CC), and recessive (TT *vs*. TC/CC) models, respectively. The Z test was performed to estimate the statistical significance of the pooled effect. The heterogeneity was evaluated by a Chi-square-based Q statistic test via *I*^2^ statistic and *P* value. No significant heterogeneity was detected if *I*^2^ < 50%, and the fixed effect model was used to calculate the pooled OR. Otherwise, the random effect model was performed. The origin of heterogeneity was detected through a subgroup analysis and a sensitivity analysis. The subgroup analysis was performed based on ethnicity, whereas the sensitivity analysis was performed by removing each article of significant heterogeneity. Publication bias was estimated using the Egger’s regression test. The statistical analysis was performed by STATA12.0 (STATA Corporation, College Station, TX). All two-tailed *P* values were computed and statistical significance cut-off was 0.05.

## Authors’ contributions

JZ and MY designed the study and extracted data, JZ and JS performed the literature search and formulated inclusion and exclusion criteria, JZ performed the statistical analysis. YB and JZ drafted the manuscript, ML revised this manuscript. All authors read and approved the final manuscript.

## Competing interests

The authors have declared no competing interests.

## Figures and Tables

**Table 1:** Descriptive characteristics of studies included in the current meta-analysis

**Ethnicity**	**Country**	**Published in**	**References**	**Diagnosis of IDD**	**Group**	**Age (year, mean ± SD)**	**Size**	**FokI alleles**		**FokI genotypes**			**HWE**
**T**	**C**	**TT**	**TC**	**CC**	***P*****value**
Caucasian	Finland	2011	[17]	Pfirrmann classification	Case	NA	81	75	87	12	51	18	0.017
Control	NA	101	82	120	17	48	36	0.883
Caucasian	Denmark	2010	[18]	No classification guideline offered	Case	13.1 ± 0.4	66	46	86	9	28	29	0.593
Control	13.1 ± 0.4	154	120	188	23	74	57	0.898
Asian	China	2007	[20]	Schneiderman classification	Case	42.7	81	75	87	12	51	18	0.017
Control	38.2	101	82	120	17	48	36	0.883
Caucasian	Italy	2012	[16]	No classification guideline offered	Case	NA	234	153	135	24	105	105	0.764
Control	NA	70	54	86	9	36	25	0.476
Hispanic	Brazil	2014	[13]	Pfirrmann classification	Case	40.0 ± 5.4 (M); 40.2 ± 5.9 (F)	121	84	158	17	50	54	0.624
Control	33.8 ± 8.2 (M); 33.8 ± 8.1 (F)	131	66	196	10	46	75	0.737
Hispanic	Mexican	2014	[15]	No classification guideline offered	Case	39.22 ± 6.88	100	95	105	15	65	20	0.002
Control	39.13 ± 6.80	100	54	86	17	51	32	0.664
Caucasian	Italy	2014	[14]	No classification guideline offered	Case	40.08 ± 9.56	267	180	354	30	120	117	0.926
Control	44.19 ± 9.11	220	163	277	32	99	89	0.601
Caucasian	Turkey	2010	[19]	Schneiderman classification	Case	NA	99	65	133	13	39	47	0.288
Control	NA	51	21	81	4	13	34	0.282

*Note:* IDD was diagnosed with magnetic resonance imaging. The exact *P* value was calculated by χ2 test. IDD, intervertebral disk degeneration; M, male; F, female; NA, not available; HWE, Hardy–Weinberg equilibrium.

**Table 2:** Association outcome between ***VDR*** FokI polymorphism and IDD

**Population**	**Test of association**				**Test of heterogeneity**		
**Model**	**OR**	**95% CI**	***P*****value**	**Model**	***P*****value**	***I*****^2^ (%)**
Pooled	Allele contrast (T *vs*. C)	1.023	0.898–1.166	0.728	R	0.016	59.2
Heterozygote (TC *vs*. CC)	1.204	0.982–1.476	0.075	R	0.024	56.6
Homozygote (TT *vs*. CC)	1.087	0.806–1.443	0.611	F	0.221	26.1
Dominant (TT/TC *vs*. CC)	1.166	0.961–1.414	0.119	R	0.022	55.4
Recessive (TT *vs*. TC/CC)	0.853	0.676–1.078	0.183	F	0.350	10.2

Caucasians	Allele contrast (T *vs*. C)	0.896	0.764–1.079	0.172	F	0.108	47.3
Heterozygote (TC *vs*. CC)	0.982	0.764–1.262	0.888	F	0.099	48.8
Homozygote (TT *vs*. CC)	0.863	0.605–1.231	0.416	F	0.424	0.0
Dominant (TT/TC *vs*. CC)	0.926	0.764–1.210	0.793	F	0.115	43.6
Recessive (TT *vs*. TC/CC)	0.879	0.735–1.050	0.156	F	0.5	0.413

Hispanics	Allele contrast (T *vs*. C)	1.395	1.059–1.836	0.018^∗^	F	0.365	0.0
Heterozygote (TC *vs*. CC)	1.254	1.049–1.498	0.013^∗^	F	0.929	0.0
Homozygote (TT *vs*. CC)	1.849	1.001–3.416	0.049^∗^	F	0.415	0.0
Dominant (TT/TC *vs*. CC)	1.742	1.174–2.538	0.006	F	0.764	0.0
Recessive (TT *vs*. TC/CC)	1.228	0.763–1.976	0.397	R	0.140	54.2

Asians	Allele contrast (T *vs*. C)	1.262	0.831–1.915	0.275	F	1	0
Heterozygote (TC *vs*. CC)	1.293	1.025–1.632	0.030^∗^	F	1	0
Homozygote (TT *vs*. CC)	1.412	0.557–3.581	0.468	F	1	0
Dominant (TT/TC *vs*. CC)	1.293	1.025–1.632	0.030	F	1	0
Recessive (TT *vs*. TC/CC)	0.880	0.446–1.735	0.712	F	1	0

*Note:* VDR, vitamin D receptor; IDD, intervertebral disk degeneration; OR, odds ratio; CI, confidence interval. F and R refer to the fixed inverse variance and random inverse variance, respectively. Significant association is indicated with asterisk.

**Table 3:** Influence analysis in allele contrast model for ***VDR*** FokI polymorphism (T ***vs.*** C)

**Ethnicity**	**Country**	**Published in**	**Ref.**	**OR**	**95% CI**
Caucasian	Finland	2011	[17]	1.058	0.843–1.330
Caucasian	Denmark	2010	[18]	1.053	0.921–1.203
Asian	China	2007	[20]	1.021	0.892–1.169
Caucasian	Italy	2012	[16]	1.062	0.928–1.214
Hispanic	Brazil	2014	[13]	1.020	0.890–1.168
Hispanic	Mexican	2014	[15]	1.093	0.956–1.251
Caucasian	Italy	2014	[14]	0.986	0.863–1.127
Caucasian	Turkey	2010	[19]	1.010	0.884–1.151

*Note:* The sensitivity of the meta-analysis was evaluated by rejecting each of the studies. OR, odds ratio; CI, confidence interval.
